# Multimodal imaging in a patient with Prader–Willi syndrome

**DOI:** 10.1186/s40942-018-0147-6

**Published:** 2018-11-30

**Authors:** Mohamed A. Hamid, Mitul C. Mehta, Baruch D. Kuppermann

**Affiliations:** Gavin Herbert Eye Institute, University of California Irvine, 850 Health Sciences Road, Irvine, CA 92697 USA

**Keywords:** Prader–Willi syndrome, Fovea plana, Macular-foveal capillaries, Type 2 macular neovascularization

## Abstract

**Background:**

Prader–Willi syndrome (PWS) is a genetic disease caused by loss of expression of the paternally inherited copy of several genes on the long arm of chromosome 15. Ophthalmic manifestations of PWS include strabismus, amblyopia, nystagmus, hypopigmentation of the iris and choroid, diabetic retinopathy, cataract and congenital ectropion uvea. An overlap between PWS and oculocutaneous albinism (OCA) has long been recognized and attributed to deletion of OCA2 gene located in PWS critical region (PWCR).

**Case report:**

A 30-year-old male patient with PWS presented with vision loss in his left eye. His right eye had normal visual acuity. Multimodal imaging revealed absence of a foveal depression and extremely reduced diameter of the foveal avascular zone in the right eye and an inactive type 2 macular neovascular lesion in the left eye.

**Conclusions:**

We report a presumed association of fovea plana and choroidal neovascularization with PWS. The use of multimodal imaging revealed novel findings in a PWS patient that might enrich our current understanding of the overlap between PWS and OCA.

## Background

Prader–Willi syndrome (PWS) is a complex genetic disorder caused by lack of expression of the paternally-inherited PWS critical region (PWCR) of chromosome 15q11.2-q13. It affects 1:10,000–1:30,000 live births. The clinical spectrum of PWS varies widely among different patients and evolves throughout their development [1]. Diagnostic hallmarks include infantile hypotonia and global developmental delay. Excessive eating during childhood usually results in central obesity which, if uncontrolled, predisposes 25% of patients to develop type 2 diabetes mellitus (DM) later in life. Mild intellectual impairment, hypogonadism and sleep apnea have been also associated with PWS. Patients often exhibit characteristic facial features such as almond-shaped up-slanted palpebral fissures, thin upper lip and small mouth with down-turned corners [2].

Ocular abnormalities reported in the context of PWS include strabismus, refractive errors, abnormal stereopsis, amblyopia and nystagmus [1]. Hypopigmentation of the iris and choroid, diabetic retinopathy, cataract, congenital ocular fibrosis syndrome and congenital ectropion uvea have also been reported [3]. Table 1 summarizes intraocular findings reported by previous publications in the context of PWS [2–29]. Herein, we report novel ocular findings in a case of PWS using multimodal imaging.

**Table 1:** Comparison between selected case series and case reports which documented ocular abnormalities in PWS patients

Study^a^	Year of publication	No. of cases	Ocular findings
Gillessen-Kaesbach et al.	1995	167	Strabismus, refractive errors, iris/fundus hypopigmentation
Butler et al.	1989	56	Strabismus, nystagmus, iris/fundus hypopigmentation
Hered et al.	1988	46	Strabismus, refractive errors, iris/fundus hypopigmentation
Butler et al.	1986	39	Iris/fundus hypopigmentation
Sanjeeva et al.	2017	34	Strabismus, refractive errors
Wiesner et al.	1987	29	Iris/fundus hypopigmentation
Spritz et al.	1997	28	Iris/fundus hypopigmentation
Fox et al.	1999	27	Strabismus, refractive errors, iris/fundus hypopigmentation
Apkarian et al.	1989	14	Strabismus, nystagmus, abnormal VEP
Roy et al.	1992	12	Strabismus, nystagmus, foveal hypoplasia, VF defects, cataract
Hittner et al.	1982	9	Strabismus, iris/fundus hypopigmentation
Creel et al.	1986	6	Strabismus, nystagmus, iris/fundus hypopigmentation, foveal hypoplasia, abnormal VEP
Kalpakian et al.	1986	1	Strabismus, CFEOM
Libov et al.	1994	1	Strabismus, refractive errors, iris/fundus hypopigmentation, glaucoma
Wang et al.	1995	1	RPE changes, cataract
Hori et al.	2012	1	PDR, cataract
Wallis et al.	1989	1	Strabismus, refractive errors, nystagmus, iris/fundus hypopigmentation
Parcheta et al.	1987	1	Iris/fundus hypopigmentation
Hayashi et al.	1992	1	Strabismus, iris/fundus hypopigmentation, foveal hypoplasia
Watanabe et al.	2006	1	PDR, cataract
Shohat et al.	1990	1	Cone degeneration
Bassali et al.	1997	1	PDR
Savir et al.	1974	1	PDR
Hattori et al.	1985	1	PDR
Futterweit et al.	1986	1	Iris/fundus hypopigmentation, glaucoma, VF defects, ectropion uveae
Gerding et al.	2012	1	RPE changes, abnormal VEP
Lee et al.	1994	1	Iris/fundus hypopigmentation
Ritch et al.	1984	1	Glaucoma, ectropion uveae

^a^Studies are arranged in a descending order according to the number of cases included. *PWS* Prader–Willi syndrome, *VEP* visually-evoked potential, *VF* visual field, *CFEOM* congenital fibrosis of the extraocular muscles, *PDR* proliferative diabetic retinopathy

## Case report

A 30-year old male patient was referred to our tertiary retina clinic at the University of California Irvine for evaluation of a macular lesion and long-standing vision drop in his left eye. He had a diagnosis of PWS. He was on insulin therapy for type 2 diabetes mellitus, amlodipine for hypertension and testosterone replacement therapy for hypogonadism. He had a history of strabismus surgery in his left eye at the age of 2 years.

His best-corrected visual acuity (BCVA) was 20/20 in his right eye and 20/150 in his left eye. Intraocular pressure was 16 mmHg in both eyes. He had full visual fields on confrontation. His eyes were orthophoric with full ocular motility in all cardinal directions and no nystagmus. Anterior segment examination was unremarkable. No iris transillumination was noted.

Fundus examination of both eyes revealed mild hypertensive retinopathy and mild nonproliferative diabetic retinopathy (NPDR). His left fundus showed a subfoveal disciform scar surrounded by a large area of pigmentary disturbance and mottling.

Green (532 nm) fundus autofluorescence (FAF) imaging revealed normal FAF in the right eye, while the left eye showed an area of central decreased FAF surrounded by a ring of increased FAF, which was, in turn, surrounded by an area of decreased FAF. This triple zone corresponds to the disciform scar and surrounding areas of retinal pigment epithelial disturbance and atrophy. There was a large area of mildly increased FAF surrounding the triple zone and occupying almost the entire macula. The latter area most likely corresponded to diseased retinal pigment epithelium (RPE) and is suggestive of prior presence of subretinal fluid.

Fluorescein angiography (FA) of both eyes showed scattered microaneurysms. The left eye showed staining of the disciform scar, but no leakage was detected.

Spectral-domain OCT (SD-OCT) of the right eye showed a shallow rudimentary foveal depression, incursion of inner retinal layers, widening of outer nuclear layer (ONL) and lengthening of outer segments; consistent with grade 1 foveal hypoplasia (fovea plana). The left eye had dome-shaped subretinal hyperreflective material (SHRM) beneath the fovea, causing disorganization of the overlying retinal layers. A few tiny cysts were noted overlying the SHRM.

SD-OCT angiography (OCTA) imaging of the right eye demonstrated a very small foveal avascular zone (FAZ) area and the presence of macular foveal capillaries (MFC) crossing the FAZ on the superficial capillary plexus (SCP) slab, and a slightly wider, but still reduced, FAZ on the deep capillary plexus (DCP) slab. OCTA of the left eye showed a type 2 macular neovascular lesion.

**Fig. 1:**
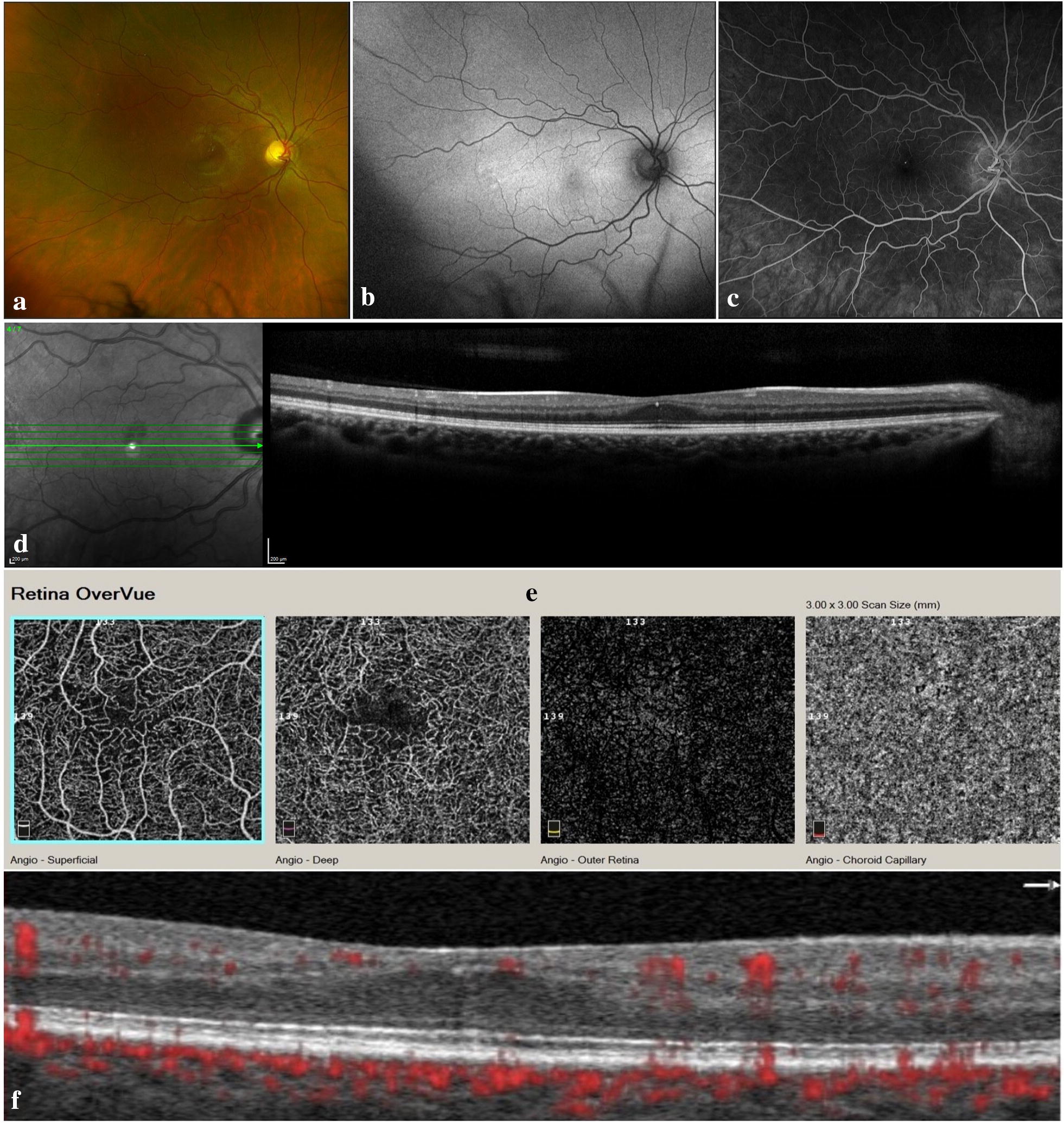
Multimodal imaging of right eye. **a** Color fundus photography. Normal fundus pigmentation. There is arteriolar tortuosity, opacification (copper or silver wiring) of the arteriolar wall and mild AV nicking consistent with mild hypertensive retinopathy. There are scattered microaneurysms consistent with mild nonproliferative diabetic retinopathy (NPDR). **b** Green fundus autofluorescence. Normal FAF. **c** Fluorescein angiography, mid arteriovenous phase. Microaneurysms appear as hyperfluorescent dots. **d** SD-OCT (horizontal scan). The shallow rudimentary foveal depression, incursion of inner retinal layers, widening of outer nuclear layer (ONL) and lengthening of outer segments are consistent with grade 1 foveal hypoplasia (fovea plana). **e** En-face OCTA slabs of superficial capillary plexus, deep capillary plexus, outer retina and choriocapillaris (from left to right). The SCP slab shows a very small FAZ area and the presence of macular foveal capillaries crossing the FAZ. The DCP slab shows reduced FAZ area, but wider than SCP FAZ. Outer retina and choriocapillaris slabs are normal and show no neovascularization. **f** B-scan structural OCT with angiographic overlay shows fovea plana and the presence of flow signals (red) in the foveal region at the levels of both SCP and DCP

**Fig. 2:**
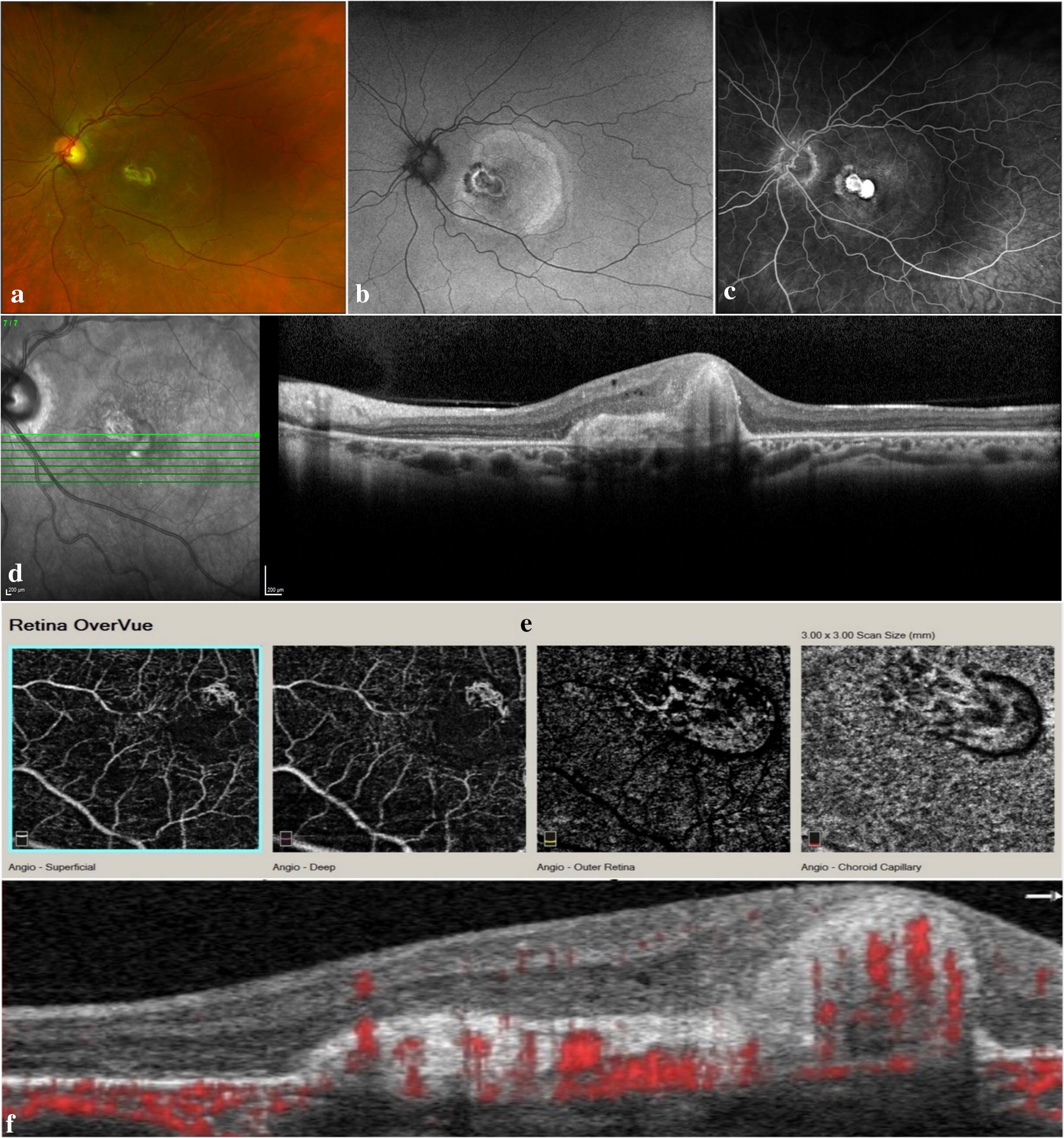
Multimodal imaging of left eye. **a** Color fundus photography. Normal fundus pigmentation. There are similar hypertensive and diabetic changes to the right eye. There is a subfoveal disciform scar surrounded by an area of pigmentary disturbance occupying almost the entire macula. **b** Green fundus autofluorescence. The fovea shows a central zone of decreased FAF surrounded by a ring of increased FAF, which is surrounded by an area of decreased FAF. This triple zone corresponds to the disciform scar and surrounding areas of RPE disturbance and atrophy. The triple zone is surrounded by a larger area of mildly increased FAF occupying almost the entire macula, corresponding to diseased RPE and suggestive of prior presence of subretinal fluid. **c** Fluorescein angiography, mid arteriovenous phase. Microaneurysms appear as hyperfluorescent dots. There is a hyperfluorescent lesion at the fovea that shows intense staining, but not leakage, consistent with scar tissue. The lesion is surrounded by a hypofluorescent rim due to blockage by pigment, which is surrounded by a large area of mild hyperfluorescence representing a window defect. **d** SD-OCT (horizontal scan). There is subretinal hyperreflective material, part of which occupies almost the entire thickness of the foveal region. There are tiny cysts present mainly in the inner nuclear layer and 1 cyst in the ganglion cell layer. **e** En-face OCTA slabs of superficial capillary plexus, deep capillary plexus, outer retina and choriocapillaris (from left to right). There is a large type 2 macular neovascular complex originating deep in the choroid and extending into the innermost retinal layers. The lesion is noted mainly on the outer retina and choriocapillaris slabs, and the top part of the lesion appears as a small neovascular tuft on the SCP and DCP slabs. **f** B-scan structural OCT with angiographic overlay shows flow signals (red) within the subretinal hyperreflective material consistent with type 2 neovascularization

Figures 1 and 2 show multimodal imaging findings in the right and left eyes, respectively.

## Discussion and conclusions

PWS results from the loss of expression of the paternally inherited copy of several contiguous genes within the PWCR on the long arm of chromosome 15. Three molecular mechanisms are implicated in the pathogenesis of PWS: Paternal deletion, maternal uniparental disomy (UPD) 15 and imprinting defect (ID). Based on clinical suspicion, diagnosis is typically confirmed by DNA methylation analysis which has a 99% sensitivity for detecting PWS caused by all 3 mechanisms, although it cannot differentiate between them. Further genetic testing is required to detect the underlying molecular mechanism and provide appropriate genetic counseling [30–32].

About 65–75% of PWS cases result from an interstitial deletion on the paternal allele of 15q11.2-q13. One of the genes in this region is *OCA2* (previously known as *P*) which is associated with type 2 (tyrosinase-positive) oculocutaneous albinism (OCA). Involvement of *OCA2* by deletion has been linked to the hypopigmentation observed in 30–50% of PWS patients [33]. PWS patients with deletion are more likely to develop hypopigmentation compared to UPD patients [34]. On the other hand, 1% of type 2 OCA patients have comorbid PWS [35]. Abnormal or absent foveal reflex has been reported in PWS patients, and considered to express the overlap between PWS and OCA [4]. A case report of 1 patient with PWS utilized SD-OCT imaging, and demonstrated only slight shallowing of the foveal depression [10]. Another report by Shohat et al. found severe cone dystrophy in a girl with PWS who had rearrangement of chromosome 15 with an increase in band 15q12. This finding might point to a possible association between this area and retinal development [24].

Excavation of the foveal pit usually begins at the 25th week of gestation and terminates 15–45 months after birth. Interruption of this process results in foveal hypoplasia [36]. Foveal hypoplasia in albinism has been postulated to result from defective melanin-bearing cells in the developing retina, which, in turn, leads to abnormal regulation of the patterns of axonal growth and pathfinding during embryogenesis [4].

The term ‘fovea plana’ (FP) was coined by Marmor et al. as a mere anatomical descriptor for the lack of a foveal pit, to emphasize the fact that the absence of a foveal depression is not concomitant with poor visual acuity or lack of cone specialization. Conversely, the term ‘foveal hypoplasia’ has been traditionally linked to poor visual function in several pathologic conditions, and often implies that the absence of the foveal depression is the culprit in abnormal vision [37]. The incidence of FP in normal eyes in a pediatric population has been estimated to range from 1.7 to 3% [38].

Subsequently, Thomas and colleagues developed an OCT-based grading system for foveal hypoplasia that reflected the different stages of foveal development. They demonstrated progressively declining visual acuity in parallel to advancing structural grade, which is a surrogate for arrest of foveal development at an earlier stage. Our patient had a shallow rudimentary foveal pit, lengthening of outer segments and widening of the ONL, consistent with grade 1 foveal hypoplasia [39].

The term “macular foveal capillaries” has been used to describe the complete or partial absence of the physiologic FAZ which is crossed by intraretinal vascular nets that communicate with the surrounding retinal capillary networks. The normal FAZ measures 500–600 microns in diameter, and shows considerable variation in size and shape among the normal population [40].

Absence of the FAZ, as determined by FA, has been reported in individuals with normal visual acuity, and in a multitude of ocular diseases including albinism, aniridia, achromatopsia, nanophthalmos and retinopathy of prematurity [41–43]. OCTA is a relatively new, non-invasive, depth-resolved method of imaging the retinal vasculature. In their recent case series, Cicinelli and colleagues described the presence of MFC in 12 eyes of 10 patients. Associated conditions included macular pucker, post-surgical macular edema, diabetic retinopathy (DR), age-related macular degeneration (AMD), dome-shaped macula (DSM), branch retinal artery occlusion (BRAO) and chronic central serous chorioretinopathy (CSCR). Five eyes in their study had loss of foveal depression due to tractional causes, but only 4 eyes lacked a normal foveal depression on SD-OCT with good visual acuity. They considered these cases to represent FP [40]. Another study used OCTA to study foveal hypoplasia in 6 patients. Two patients had a diagnosis of OCA, while lack of a foveal depression was an incidental finding in 4 patients, all of which had a good visual acuity and an otherwise normal retina. The FAZ was completely absent in the SCP and only partially so in the DCP [44]. This is similar to the findings we observed in our patient who had almost absent FAZ in SCP and a markedly reduced FAZ in DCP, but still larger than in SCP. Other small case series and case reports have described similar findings using OCTA [45–47].

An inverse correlation between FAZ area and central macular thickness (CMT) and volume (CMV) has been demonstrated using OCTA. As CMT and CMV increase, foveal pit depth, volume and FAZ size decrease [48]. Another study utilizing FA and SD-OCT showed the FAZ area to inversely correlate to foveal pit depth, diameter and volume [49]. These findings emphasize the role FAZ purportedly plays in foveal pit development during primate embryogenesis.

Since the development of FAZ predates that of foveal excavation, it is plausible that the FAZ area impacts the centrifugal migration of inner retinal layers, and, hence, foveal pit morphology [50]. However, neither appears to be instrumental in determining visual function, as evidenced by the substantial overlap between foveal pit morphology in the normal population and in patients with albinism, and the lack of correlation between visual function and pit depth [49]. It is more likely that other features are more influential, such as foveal cone packing, elongation of outer segments and lengthening of Henle’s fibers. None of these features appears to be dependent on foveal pit morphology for their characterization [51].

Several causes of choroidal neovascularization (CNV) have been reported in individuals younger than 50 years. These include pathologic myopia, angioid streaks, chronic CSCR, type 2 macular telangiectasia, choroidal rupture, posterior uveitis, choroidal tumors, hereditary retinal dystrophies and optic nerve head anomalies [52]. When there is no apparent cause for neovascularization, it is termed ‘idiopathic’. Idiopathic CNV is usually unilateral and has better visual prognosis than CNV secondary to age-related macular degeneration (AMD) [53].

To the best of our knowledge, this finding has not been reported in the context of PWS. Our patient was not highly myopic. A possible explanation for CNV development is silencing of *NDN* gene, which is a maternally imprinted gene that maps to the PWCR and encodes for necdin. Necdin is a protein that plays a role in neuronal differentiation and development, and is thought to have tumor-suppressing and anti-angiogenic properties [54]. However, confirmation of this hypothesis requires complex genetic testing and an in-depth investigation of a cause-effect relationship. Alternatively, the CNV could be the result of unreported old trauma or posterior uveitis that resulted in chorioretinal scarring and an RPE defect. It is also possible that this is a mere coexistence of an idiopathic CNV.

Our patient displayed many characteristic features of PWS, including short stature, typical facial dysmorphism, hypogonadism and type 2 DM. He also had a history of prior strabismus surgery. He was overweight with a body mass index (BMI) of 34. There was no hypopigmentation of his skin, hair or eyes. This could be explained by the relatively old age of the patient, since patients with OCA are known to exhibit progressive darkening of skin, hair and eyes with age, and hypopigmentation in PWS patients is usually less severe than that noted with OCA patients [55].

In summary we report novel ocular findings in a PWS patient using multimodal imaging. These are fovea plana and macular foveal capillaries in 1 eye and type 2 macular neovascularization in the other eye. The patient was followed for 1 year, during which examination and multimodal imaging were repeated twice and showed the same findings each time. Based on clinical stability and absence of leakage from the neovascular lesion, the decision was made to observe the patient closely and not to administer anti-angiogenic therapy. The patient was counseled on tight control of his blood glucose level and blood pressure to prevent further progression of his fundus diabetic and hypertensive changes.

## Abbreviations

AMD: age-related macular degeneration
BCVA: best-corrected visual acuity
BMI: body mass index
BRAO: branch retinal artery occlusion
CMT: central macular thickness
CMV: central macular volume
CNV: choroidal neovascularization
CSCR: central serous chorioretinopathy
DCP: deep capillary plexus
DM: diabetes mellitus
DR: diabetic retinopathy
DSM: dome-shaped macula
FA: fluorescein angiography
FAF: fundus autofluorescence
FAZ: foveal avascular zone
FP: fovea plana
ICGA: indocyanine green angiography
ID: imprinting defect
MFC: macular foveal capillaries
NDN: necdin
nm: nanometer
NPDR: nonproliferative diabetic retinopathy
OCA: oculocutaneous albinism
OCTA: optical coherence tomography angiography
ONL: outer nuclear layer
PDR: proliferative diabetic retinopathy
PWCR: Prader–Willi critical region
PWS: Prader–Willi syndrome
RPE: retinal pigment epithelium
SCP: superficial capillary plexus
SD-OCT: spectral domain optical coherence tomography
SHRM: subretinal hyperreflective material
UPD: uniparental disomy
VEP: visually-evoked potential
VF: visual field
